# Serum leptin, a potential predictor of long‐term angiographic progression in Takayasu’s arteritis

**DOI:** 10.1111/1756-185X.13718

**Published:** 2019-10-09

**Authors:** Lili Ma, Wensu Yu, Xiaomin Dai, Mengmeng Yin, Yujiao Wang, Ying Sun, Xiufang Kong, Xiaomeng Cui, Sifan Wu, Zongfei Ji, Lingying Ma, Huiyong Chen, Jiang Lin, Lindi Jiang

**Affiliations:** ^1^ Department of Rheumatology Zhongshan Hospital Fudan University Shanghai China; ^2^ Evidence‐based medicine center Fudan University Shanghai China; ^3^ Department of Radiology Zhongshan Hospital Fudan University Shanghai China

**Keywords:** disease progression, leptin, radiography, Takayasu's arteritis

## Abstract

**Aim:**

In patients with Takayasu's arteritis (TA), current biomarkers that properly reflect the progression of the vascular structure remain absent. We aimed to determine the serum leptin level to investigate its relationship with imaging changes and assess its value as a predictor for long‐term radiological progression.

**Method:**

This study included 34 untreated TA patients and 40 age‐matched healthy controls. At baseline and during the 5‐year follow‐up, we assessed disease activity using Kerr's criteria and Indian Takayasu Clinical Activity Score (ITAS2010) and monitored laboratory biomarkers as well as imaging findings. Serum leptin levels were measured by enzyme‐linked immunosorbent assay.

**Results:**

The baseline serum leptin levels were significantly higher in TA patients than in healthy controls. Leptin was significantly positively correlated with triglyceride and high‐density lipoprotein cholesterol levels and negatively correlated with fibrinogen and C‐reactive protein levels. Patients were subdivided into three groups based on their baseline leptin level. During a 5‐year follow‐up, patients in the high and medium leptin groups showed more radiological progression compared to those in the low leptin group. Cox proportional hazard regression analysis showed that a high serum leptin level was a positive predictor of radiological progression.

**Conclusion:**

Leptin is a potential biomarker for assessing TA structural progression. Untreated patients with elevated serum leptin levels are at a higher risk of progression in the aorta. Thus, the leptin level can be a predictor of long‐term radiological progression.

## 1 INTRODUCTION

Takayasu arteritis (TA) is an autoimmune disease characterized by granulomatous inflammation of the all three layers of the aorta and its branches. Stenosis, occlusion, lumen dilation, and vessel wall thickening are substantial characteristics of vessel damage. Disease activity is a major concern in clinical practice and is assessed using inflammatory biomarkers such as erythrocyte sedimentation rate (ESR) and serum C‐reactive protein (CRP), as well as Kerr Criteria established by National Institutes of Health in 1994 and Indian Takayasu Clinical Activity Score (ITAS2010).^1^ Vessel wall structure is currently examined by non‐invasive imaging methods, such as magnetic resonance angiography (MRA), computed tomographic angiography (CTA), color Doppler ultrasound (CDU), and positron emission tomography‐CT (PET‐CT).^2^, ^3^, ^4^, ^5^ In particular, MRA merits preference in the assessment of vessel damage progression because it affords the unique advantage of real‐time angiography in TA. Nevertheless, biomarkers that can predict radiological progression of TA remain to be elucidated.

The etiology of TA is still unknown, although previous studies have shown that both innate and adaptive immune responses, as well as cytokines and chemokines, are involved.^6^, ^7^, ^8^, ^9^ A study conducted in Japan suggested that serum levels of monoclonal anti‐tumor necrosis factor‐α (TNF‐α) antibody might be a potent biomarker that can reflect disease activity.^10^ Our previous studies showed that interleukin‐6 (IL‐6) was not only a strong marker for active disease but also played a critical role in pathogenesis and vascular fibrosis.^7^, ^11^ IL‐18 levels have also been reported to be significantly higher in active patients assessed using ITAS2010. However, few studies have concentrated on the involvement of these cytokines and chemokines in vascular remodeling or their utility for predicting structural progression in TA. Local expression of IL‐17 in active arterial infiltration seemed controversial in various studies.^7^, ^12^ Importantly, even TA patients in apparent remission state may show elevated levels of many inflammatory cytokines such as IL‐17 with extensive disease or previous ischemic events, indicating that latent but progressive arterial damage can persist irrespective of conventional treatments and alleviated symptoms.^13^ Thus, it is urgent to discover proper biomarkers of vascular abnormalities for subsequent imaging examinations.

Adipose tissue (AT) was once considered as merely supportive tissue, but its incredible role in metabolism and inflammation have been revealed in recent years.^14^, ^15^ Perivascular AT (PVAT) directly surrounds the outer vascular wall without separation of the fascial plane; therefore, its secretions, called adipokines, allow for the local regulation of vascular function.^16^ Leptin is the first discovered and the most studied adipokine. As a hormone, it maintains energy homeostasis and controls body weight.^17^, ^18^ As a cytokine, it mostly works as an immune enhancer together with other proinflammatory factors including IL‐6 and TNF‐α.^19^ The level of leptin is thought to correlate closely with immunity. More precisely, a high concentration of leptin in the circulating blood makes individuals susceptible to rheumatic and metabolic diseases, while a low concentration suppresses the function of immune cells, leading to frequent infections.^20^, ^21^ The general effects of leptin are attributable to leptin receptors (LEPRs) on the membranes of various types of cells, notably immune cells. As leptin binds to the long isoform of LEPRs, the LEPR homodimer automatically forms, which subsequently activates the Janus‐activated kinase (JAK) and signaling transducer and activator of transcription (STAT) pathways.^22^, ^23^

Thus far, there are few studies focusing on the role that leptin plays in TA. In this study, we aimed to determine the expression level of leptin, in order to investigate its relationship with the progression of vascular damage and assess its predictive value for long‐term radiological progression.

## 2 METHODS AND MATERIALS

### 2.1 Study subjects

We included 34 TA patients who visited the Department of Rheumatology, Zhongshan Hospital, Fudan University (Shanghai, China) for the first time from January 2010 to December 2012. All the patients were classified according to the 1990 American College of Rheumatology classification criteria, and none of them had undergone any previous treatment.^24^ Forty age‐matched female volunteers were enrolled as healthy controls during the same period. No subject in this study was obese or had hyperlipidemia or diabetes mellitus. The study protocol was approved by the Ethics Committee of Zhongshan Hospital (Approval No. B2013‐115[3]). All patients provided their written informed consent for inclusion in this study.

### 2.2 Follow‐up

All 34 patients were suggested a 5‐year follow‐up to observe their condition in a dynamic view, and the ideal interval was 3‐6 months. The primary endpoint in this study was progression of arterial lesions detected on a radiological examination (see definition in Assessment of disease activity). Alteration of dosage or drugs was performed if clinical presentation and results of work‐ups indicated either alleviation or exacerbation. Radiological examinations were suggested at least once annually.

### 2.3 Assessment of disease activity

Demographic characteristics of the patients, as well as their follow‐up data including clinical presentation, laboratory indexes, radiological images, and reports, were stored in the database of Zhongshan Hospital. We assessed disease activity using Kerr's criteria, ITAS2010, at least one kind of radiological examination, and acute‐phase reactants. In this study, new onset or worsening of two or more characteristics in Kerr's criteria, or no less than 2 scores in ITAS2010 after ruling out infectious disease indicated "active disease".^1^, ^25^ Patients who remained in active disease at the end of the induction period were considered as refractory patients, and those with previous remission for over 6 months were considered as relapse when active disease was documented using Kerr's criteria by an experienced rheumatologist.

Radiological examinations included MRA, contrast‐enhanced ultrasound (CEU) and CTA. We assessed the arterial involvement in these TA patients by evaluating their imaging presentations. Here we concentrated on the following changes in vessels: stenosis, occlusion, dilation, aneurysm formation, and thickening of the wall. We defined radiological progression if any of the following findings were positive: (a) new artery involvement shown by any of the radiological procedures including MRA, CTA, and CEU; and (b) worsening of stenotic arteries, including newly emerged occlusions and no less than 20% narrowing of the lumen.

### 2.4 Measurement of leptin and acute‐phase reactants

Venous blood samples obtained from TA patients and healthy controls were centrifuged (relative centrifugal force: 290 *g*, and centrifugal time: 15 minutes) and supernatant specimens were separated and stored at −80°C until assayed. The level of leptin (ALPCO) in serum specimens (for all TA patients and controls) was measured using enzyme‐linked immunosorbent assay (ELISA) kits.

### 2.5 Statistics

All statistical data were analyzed with the Statistical Package for the Social and Sciences (SPSS, version 21.0; SPSS Inc). Results were expressed as mean ± SD values. Hazard ratios (HRs) and 95% confidence intervals (95% CIs) were calculated for each factor by Cox proportional hazards regression analysis. Kaplan–Meier survival curve, drawn by GraphPad Prism 7 (GraphPad), was used to depict the occurrence of radiological progression during the 5‐year follow‐up. Mann‐Whitney *U* test, Chi‐square test, Kruskal‐Wallis test, and Spearman's rho were used for comparison of data. Differences were considered significant at *P* < .05.

## 3 RESULTS

### 3.1 Baseline information of TA patients

The average age at onset of TA symptoms was 30 ± 12 years (range, 11‐55 years). Median disease duration in TA patients was 12 months (range, 1‐360 months). All 34 patients were considered TA active on the basis of Kerr's criteria, while only 21 were classified as active using ITAS2010. According to the Hata and Numano angiographic classification, four patients (11.8%) were classified as having type 1 involvement, 4 (11.8%) as type 2a, 4 (11.8%) as type 2b, 6 (17.6%) as type 3, 3 (8.8%) as type 4, and 13 (38.2%) as type 5.

Thirty‐four patients underwent MRA or CTA examinations at Zhongshan Hospital or provided valid reports from other clinical centers at the beginning of the study. All 34 patients showed the involvement of at least one artery in their imaging reports: 7 (20.6%) showed vessel occlusion, 11 (32.4%) showed dilation of the lumen(s) or aneurysm formation, and 18 (52.9%) presented with thickening of the vessel wall.

Twenty‐seven patients (79.4%) started glucocorticoid (GC) therapy (55.0 ± 33.7 mg, converted to prednisone potency), and eight of them received methylprednisolone while the rest were initially on prednisone. For additional immunosuppression, 19 patients (55.9%) received cyclophosphamide, 14 (41.2%) were administered hydroxychloroquine, 2 (5.9%) each received azathioprine and thalidomide, 1 (3.0%) was on methotrexate, and 4 (10.8%) received biological agents. Five patients (14.7%) were not taking any medication at the beginning.

### 3.2 Higher levels of leptin in TA patients than in the controls

The baseline serum levels of leptin were significantly higher in TA patients (24.4 ± 15.8 ng/mL) than in healthy controls (7.2 ± 5.0 ng/mL) (*P* < .001). Patients were divided into three groups on the basis of serum leptin levels, namely high (11 patients; range, 28.9‐66.9 ng/mL), medium (11 patients; range, 17.4‐28.8 ng/mL), and low (12 patients; range, 0.8‐17.3 ng/mL). The baseline information of the three leptin subgroups is listed in Table 1.

**Table 1:** The baseline characteristics of the 3 leptin subgroups

	High group N = 11	Medium group N = 11	Low group N = 12	*P* value
Basic information				
Onset age, y	32.5 ± 13.3	22.3 ± 4.1	34.8 ± 12.7	.049*
Duration, mo	97.20 ± 122.53	64.50 ± 79.57	14.75 ± 19.07	.268
Female, n (%)	11 (100%)	10 (90.9%)	9 (75%)	.177
Biomarker level				
ESR, mm/h	41.18 ± 34.33	42.09 ± 23.71	60.92 ± 37.66	.272
CRP, mg/L	15.6 ± 21.19	17.45 ± 16.44	43.45 ± 43.83	.130
PLT, /10^9^ L	269.11 ± 93.32	270.78 ± 68.48	303.09 ± 94	.619
FIB, mg/L	352.88 ± 175.98	339.63 ± 143.64	456 ± 114.31	.124
TC, mmol/L	4.48 ± 0.94	3.99 ± 1.23	3.56 ± 0.66	.169
TG, mmol/L	1.40 ± 0.58	1.06 ± 0.32	0.90 ± 0.23	.108
HDL, mmol/L	2.44 ± 0.73	1.99 ± 0.86	1.72 ± 0.31	.074
LDL, mmol/L	1.42 ± 0.57	1.53 ± 0.77	1.54 ± 0.53	.895
Hb, g/L	117.82 ± 12.39	123.88 ± 17.7	117.44 ± 18.56	.745
IgG, g/L	14.72 ± 5.56	11.9 ± 4.24	13.14 ± 5.95	.562
IgM, g/L	1.46 ± 0.66	1.58 ± 0.64	1.36 ± 0.56	.870
IgA, g/L	2.8 ± 1.37	2.43 ± 1.29	2.56 ± 1.23	.874
IgE, IU/mL	41 ± 47.93	100.5 ± 161.85	276.6 ± 416.05	.296
Globulin, g/L	32.4 ± 6.83	29.18 ± 7.26	34.21 ± 6.78	.535
Albumin, g/L	38 ± 3.27	38.51 ± 3.53	36.32 ± 4.45	.319
Prescription				
GC, n (%)	8 (72.7%)	9 (81.8%)	10 (83.3%)	.803
IS, n (%)	10 (90.9%)	10 (90.9%)	9 (75%)	.468
Disease activity				
ITAS2010 score	1.45 ± 1.51	3.27 ± 3.13	3.17 ± 2.59	.233
Kerr score	2.45 ± 0.52	2.82 ± 0.75	2.83 ± 0.58	.287
Vascular damage				
Stenosis, n	9	10	7	.142
Occlusion, n	3	4	0	.096
Dilation and aneurysm, n	1	5	5	.109
Thickening, n	8	5	5	.146
Occlusion rate	0.1 1 ± 0.24	0.19 ± 0.26	0 ± 0	.108

Note

Data shown as mean ± SD unless otherwise indicated.

* *P *< .05

Abbreviations: CRP, C‐reactive protein; FIB, fibrinogen; ESR, erythrocyte sedimentation rate; GC, glucocorticoid; Hb, hemoglobin; HDL, high‐density lipoprotein; Ig, immunoglobulin; IS, immunosuppressant; ITAS2010, Indian Takayasu Clinical Activity Score 2010; LDL, low‐density lipoprotein; PLT, platelet; TC, total cholesterol; TG, triglyceride.

Interestingly, patients with type 2b showed significantly higher leptin levels than that of other types (40.4 ± 17.9 ng/mL, 21.7 ± 14.0 ng/mL, respectively, *P* = .029). However, as for Kerr score and ITAS2010 score, there were no significant differences in these types.

### 3.3 Correlation between baseline levels of leptin and other substances

We found a significant positive correlation between leptin and triglyceride as well as high‐density lipoprotein (HDL) levels (*r* = .421, *P* = .046; *r* = .432, *P* = .040, respectively). Significant negative correlation was observed between leptin and fibrinogen as well as CRP levels (*r* = −.383, *P* = .049; *r* = −.343, *P* = .05, respectively). However, ESR, as a common inflammation‐indicating index, was not as well correlated as CRP with leptin (Table 2). The levels of hemoglobin (Hb), albumin, and globulin showed poor relationships with leptin levels.

**Table 2:** Correlation between leptin and other substances at baseline

	FIB	PLT	CRP	ESR	TG	TC	HDL	LDL
*r*	−.383	−.186	−.343	−.285	.421	.353	.432	−.190
*P*	.049*	.333	.05*	.102	.046*	.091	.040*	.386

Abbreviations: CRP, C‐reactive protein; ESR, erythrocyte sedimentation rate; FIB, fibrinogen; HDL, high‐density lipoprotein; LDL, low‐density lipoprotein; PLT, platelet; TC, total cholesterol; TG, triglyceride.

* *P* < .05

### 3.4 Five‐year follow‐up details

**Figure 1:**
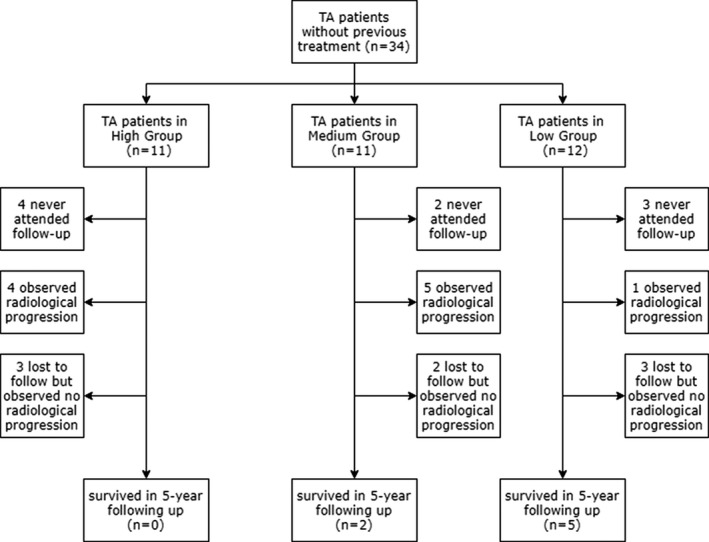
Flow diagram of 5‐year follow‐up with observed events

The follow‐up period was 4.9 ± 0.7 years, slightly adjusted by the visit frequency of each patient. Ten patients (29.4%) showed progression throughout the radiological measurements during serial visits (nine by MRA, median 2.87 years; one by CTA at 3.14 years); 8 (23.5%) remained stable or showed amelioration during this process; 9 (26.5%) did not attend follow‐up examinations (four patients in the high group, 2 in the medium group, and three in the low group); while the remaining 7 (20.6%) were lost to follow‐up before the endpoint (range, 0.20‐4.09 years; see Figure 1).

We investigated the reasons why patients failed to attend or persevere with follow‐up. In the nine patients without any follow‐up data, 5 (55.6%) chose other departments or hospitals, 3 (33.3%) were for personal reasons and 1 (11.1%) for unexplained reasons. Of note, of the patients who did not accept any drugs at the beginning, none entered the follow‐up. In the seven patients lost to follow‐up half way, 2 (28.6%) altered hospitals and the rest quit for unknown reasons.

Here we compared baseline characteristics of the “not attending follow‐up” subjects with the rest. ESR level was the only item that differed significantly between the two groups, and the averages were both higher than the upper normal limit (see Table S1).

### 3.5 Treatment and responses

After 6‐month induction, the mean dosage of GC decreased to 16.0 ± 7.6 mg (converted to prednisone potency), and the mean cumulative dosage of cyclophosphamide was 4.7 ± 2.4 g. Four subjects were diagnosed as refractory patients (1, 1, and 2 in high, medium and low groups, respectively). In their induction period, 3 (75%) consented to the combined treatment of GC and cyclophosphamide, and the other 1 (25%) accepted GC and methotrexate. Subsequently, three patients changed to leflunomide and managed to be in remission, and one was lost to follow‐up.

Relapse occurred in nine subjects (four in high, four in medium and one in low groups) at 3.13 years median (interquartile range, 1.91‐4.72 years). Six (66.7%) increased the dosage of GC, immunosuppressant, or both. Three subjects (33.3%) stopped using the original immunosuppressant. One (11.1%) was lost to follow‐up at the time of relapse, and 1 (11.1%) experienced multiple relapses during follow‐up. Interestingly, angiographic progression was not always concomitant with TA relapse, for only 3 (33.3%) showed both simultaneously, and 2 (22.2%) presented with relapse prior to progression and 4 (44.4%) showed mere relapse.

We tested the ESR and serum CRP levels in TA patients and compared the baseline levels with those obtained after the 1st‐year treatment. Eighteen patients attended a follow‐up visit at the end of the 1st year, and 17 had normal CRP levels (0‐8 mg/L) or a much lower CRP level than before (25.00 ± 26.17 mg/L at baseline, 5.37 ± 7.46 mg/L at 1 year, *P* = .033). The 1‐year ESR level decreased to the reference range (0‐20 mm/h) in 14 patients, and it was significantly lower than the baseline level (16.39 ± 15.35 mm/h and 35.47 ± 29.45 mm/h, respectively, *P* = .006).

Furthermore, we traced the ESR and serum CRP levels in TA patients during 5 years of follow‐up (Figure S1). Both ESR and CRP levels significantly decreased since TA patients accepted therapy and generally remained stable in serial visits. No significant differences were found in the three subgroups.

### 3.6 Leptin as a potential predictor in long‐term angiographic progression

Based on our previously described definition of radiological progression, we traced the latest condition of the involved vessels and compared them with the previous results. Eighteen patients consented to periodic MRA examination, and 16 to CEU and four to CTA (some patients with multiple kinds of examinations). The mean interval of radiological examination was 1.7 ± 1.4 years, and 11 patients (47.8%) managed to take the examinations annually or more frequently.

**Figure 2:**
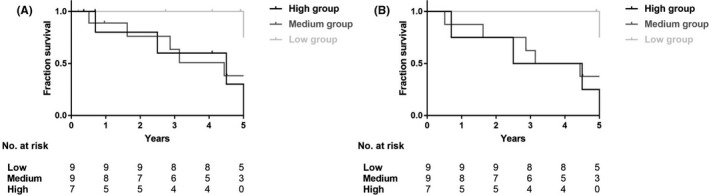
Kaplan–Meier curve of angiographic progression. Right‐censored data indicate that the subjects were lost to follow‐up at that point before any progression of arterial lesion was observed. A, Including the 7 subjects lost to follow‐up. B, After removing the 7 subjects lost to follow‐up

Six subjects in the low group finished the 5‐year follow‐up assessment, and five of them (range, 4.91‐5.36 years), containing one refractory patient and one relapsing patient, showed no progression in imaging although some of them were marked as right‐censored data in Figure 2. The frequencies of angiographic progression in the medium and high groups were much more than that in the low group. Univariate Cox proportional hazard regression analysis showed that high and medium leptin levels were both positive predictors of radiological progression (HR, 10.960 and 7.092; 95% CI, 1.195‐100.917 and 0.813‐61.858; *P* = .034 and *P* = .076, respectively). Similar results were identified in multivariate Cox proportional hazard regression analysis using explanatory variables including onset age, duration, and Kerr score (Table 3). Two patients with type 2b (50.0%) showed angiographic progression, while none of the patients with type 1 or 2a did. Moreover, type 2b failed to be a predictor (HR 1.538, 95% CI 0.315‐7.498, *P* = .594), and so did all other types.

**Table 3:** Cox proportional hazard regression analysis for angiographic progression

	Total			Complete follow‐up		
	HR	95% CI	*P* value	HR	95% CI	*P* value
HR (crude)						
Low group	1	‐	‐	1	‐	‐
Medium group	7.092	0.813‐61.858	.076	6.315	0.722‐55.235	.096
High group	10.960	1.195‐100.917	.034*	11.882	1.292‐109.285	.029*
HR (adjusted)^a^						
Low group	1	‐	‐	1	‐	‐
Medium group	7.550	0.754‐75.592	.085	10.262	0.865‐121.675	.065
High group	12.681	1.055‐152.444	.045*	21.428	1.468‐312.726	.025*

a Adjusted by onset age, disease duration and Kerr score.

* *P* < .05

## 4 DISCUSSION

The involvement of PVAT and adipokines has been proven to be crucial in some systemic diseases. In rodents, the composition of PVAT varies in different places, such as brown AT surrounding thoracic aorta and white AT surrounding mesenteric artery.^26^ In humans, PVAT is basically more akin to white AT than brown AT in morphology, and so far its heterogeneity in humans has not been studied as well as that in rodents, but a recent study of the coronary artery and internal thoracic artery hints that differential phenotypes of PVAT may lead to vascular lesions in specific areas.^27^ PVAT is characterized by small adipocytes with multiple small lipid droplets, vascular regulation, and interplay with immune cells.^28^, ^29^ The promotion of PVAT inflammation can be largely attributed to its interactive communication with macrophages. Adipocytes excrete free fatty acids to up‐regulate the expression of macrophage inflammatory genes. Thus, the hydrolysis of triglyceride to free fatty acids can also be enhanced through TNF‐α expressed by stimulated PVAT macrophages, forming a loop to evoke inflammation.^30^ PVAT inflammation participates in the development of cardiovascular diseases by breaking the homeostasis of vessels, leading to excessive secretion of proinflammatory factors such as IL‐6, IL‐17, leptin, visfatin, and TNF‐α and reduction of anti‐inflammatory factors including adiponectin, angiotensin 1‐7, and IL‐10,^31^ and many of these factors have also been reported to contribute to the pathogenesis of TA.^7^, ^10^, ^32^ To date, there is no definitive evidence showing how PVAT inflammation affects the vascular wall in TA.^33^

Leptin levels can be regulated by body mass index (BMI) and body fat mass; therefore, obese and malnourished people as well as patients with diabetes mellitus were not included so as to minimize the interference of possible confounding factors. Our results showed that serum leptin level was negatively correlated with CRP level (significant) and ESR (not significant) in pretreatment TA patients, although these substances were all highly expressed in comparison with healthy controls, which contributes to our confusion regarding the role of leptin. The relationship between leptin and non‐specific inflammatory markers appears to be controversial in other diseases as well. One study reported simultaneous elevation of ESR and leptin and CRP levels in some hemodialysis patients, but the authors suggested that it was BMI, but not the two inflammatory markers that had a significant correlation with leptin.^34^ High leptin level was also found in fibromyalgia patients without a firm relationship with ESR and CRP levels, implying its poor capacity to indicate disease activity.^35^ However, the involvement of leptin in systemic inflammation was still confirmed in the two studies above, as well as many other studies focused on rheumatoid arthritis (RA),^36^ systemic lupus erythematosus (SLE),^37^ immunoglobulin A (IgA) vasculitis^38^ and Behçet's disease.^39^ In our study, we speculate that elevated CRP levels indicate an aggravating inflammatory response, which might up‐regulate the general metabolism and eventually diminish secretion of serum leptin.

In our study, significant correlation between serum leptin and involved arteries was barely found, but none of the cases in the low group showed lumen occlusion. Leptin plays a substantial role as a mediator in the progression of atherosclerotic vessel damages such as endothelial dysfunction and neointimal hyperplasia.^40^ Moreover, local adipokine secretion including PVAT‐derived leptin significantly contributes to the initiation and expansion of coronary disease.^41^ Importantly, arterial fibrosis is one of the impressive features in the healing phase of TA,^42^ and leptin‐induced vascular extracellular matrix remodeling may eventually cause fibrosis.^43^ According to the results of Cox proportional hazard regression analysis, during our 5‐year follow‐up observation, TA patients who initially showed hyperleptinemia had a higher risk of progressive arterial damage thereafter. The initial use of GC and immunosuppressants was excluded from explanatory factors in Cox regression because of the insignificant differences in the subgroups and because another study found no significant differences in leptin levels between TA patients who accepted GC treatment and those who did not.^44^

We also found a significant correlation between leptin and triglyceride and HDL levels. Indeed, the effects of blood lipids in vascular damage should not be neglected. Hyperlipidemia has been reported to induce vascular smooth muscle cell proliferation involving Wnt/β‐catenin signaling in rats,^45^ and Wnt pathways are capable of regulating inflammation, pathological angiogenesis, and calcification of vessels.^46^ However, our results showed that serum triglyceride and HDL levels were within the reference range in most of the patients at baseline, with no significant differences among the three subgroups. Therefore, elevation of leptin levels deserves more attention as a predictor to arterial involvement.

Many rheumatologists have noted the importance of radiological manifestations in assessing TA disease activity. Non‐invasive procedures such as MRA, CTA, and CDU can accurately recognize vessel abnormalities even before the appearance of clinical symptoms and signs, but without a gold standard, the sensitivity and specificity of these methods can be highly variable in different studies.^47^ In this study, we did not merge vessel wall thickening into our definition of radiological progression for three reasons. First, the optimal threshold value of vessel wall thickness has not been determined; for instance, the cut‐off carotid intima‐media thickness (IMT) varied from 0.8 to 2 mm in various definitions partly due to heterogeneity between studies and inadequate samples.^48^, ^49^ Second, data for the utility of vascular wall enhancement on CTA and MRA in identifying active disease are conflicting.^40^ Finally, MRA can only measure three‐layer thickness but not IMT.

The main limitation of our study was its cross‐sectional nature and the lack of dynamic observations for assessment of leptin during follow‐up examinations. Some of our patients were lost to follow‐up during our 5‐year observation period. Therefore, the results demonstrated herein need to be confirmed by larger‐size samples and further studies.

## 5 CONCLUSION

In conclusion, leptin is a potential biomarker to assess TA structural progression. Untreated patients with elevated serum leptin levels at baseline faced a higher risk of progression in arteries during the 5‐year follow‐up period, and leptin levels can be a predictor of long‐term radiological progression.

## CONFLICT OF INTEREST

No conflicts of interest.

## AUTHOR CONTRIBUTIONS

LL Ma, WS Yu and LD Jiang participated in the design of this study. XM Dai contributed to ELISA samples testing. MM Yin, YJ Wang, Y Sun, XF Kong, XM Cui and SF Wu participated in follow‐up and data entry. ZF Ji contributed to data administration. LY Ma was in charge of quality assurance in the study. HY Chen and J Lin contributed to radiological assessment. LL Ma and WS Yu drafted the manuscript.
