# Supplementary figures and images for: Serum leptin, a potential predictor of long‐term angiographic progression in Takayasu’s arteritis
Source: Int J Rheum Dis. 2019 Oct 9;22(12):2134–42. doi: 10.1111/1756-185X.13718 (PMC6916353; doi:10.1111/1756-185X.13718)

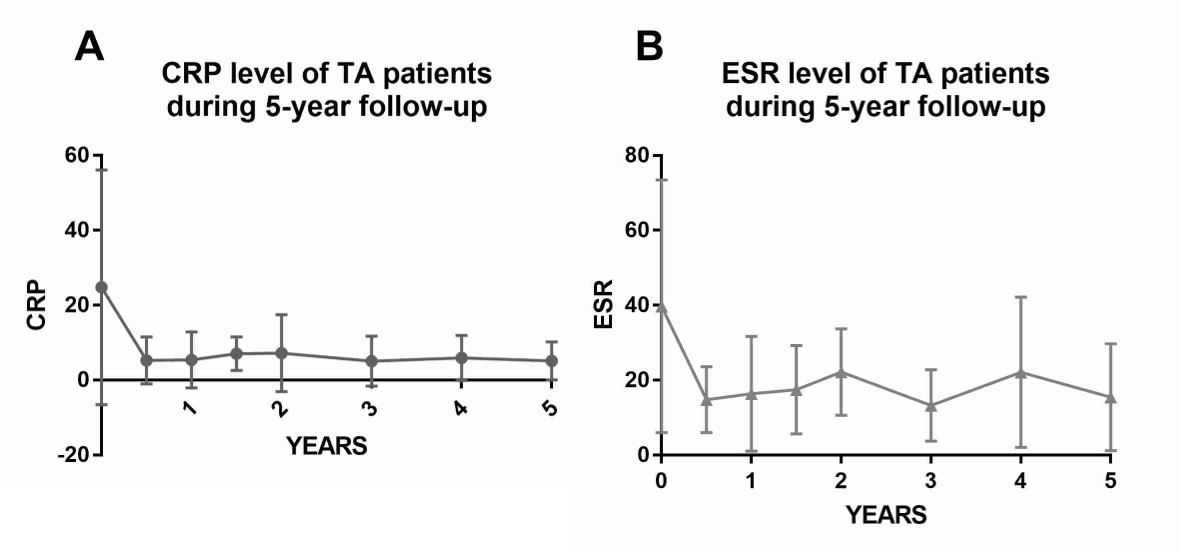

Supplement: Supplementary file 1 [file APL-22-2134-s001.jpg]
