# Supplementary material for: Serum leptin, a potential predictor of long‐term angiographic progression in Takayasu’s arteritis
Source: Int J Rheum Dis. 2019 Oct 9;22(12):2134–42. doi: 10.1111/1756-185X.13718 (PMC6916353; doi:10.1111/1756-185X.13718)
Supplement: Supplementary file 2 [file APL-22-2134-s002.docx]

Supplementary table 1

Baseline characteristics of not-attending-follow-up subjects VS the other patients

|  | **Not-attending-follow-up**  **(n=9)** | **The rest**  **(n=25)** | **P value** |
| --- | --- | --- | --- |
| **Baseline information** | | | |
| Onset age, years | 35.33±11.37 | 29.28±12.15 | 0.160 |
| Duration, months | 37.86±61.16 | 61.16±93.28 | 0.464 |
| Female, n, (%) | 8 (88.9%) | 22 (88%) | 0.969 |
| **Hata and Numano classification** | | | |
| Type 1, n | 2 | 2 |  |
| Type 2a, n | 2 | 2 |  |
| Type 2b, n | 1 | 4 |  |
| Type 3, n | 1 | 5 |  |
| Type 4, n | 1 | 2 |  |
| Type 5, n | 2 | 10 |  |
| **Leptin group** | | | |
| Low group, n | 2 | 9 |  |
| Medium group, n | 3 | 9 |  |
| High group, n | 4 | 7 |  |
| **Leptin level, ng/ml** | **26.19±15.60** | **23.80±16.19** | **0.626** |
| **Disease activity** | | | |
| Kerr score | 2.56±0.53 | 2.76±0.66 | 0.514 |
| ITAS score | 1.33±2.06 | 3.12±2.60 | 0.060 |
| **Biomarker level** | | | |
| ESR, mm/hour | 29.44±15.88 | 55.28±35.04 | 0.030 |
| CRP, mg/L | 24.07±45.15 | 26.04±25.88 | 0.290 |
| **Vascular damage** | | | |
| Stenosis^†^, Mean ± SD, (n) | 1.50±1.38 (5) | 2.08±1.38 (21) | 0.347 |
| Occlusion^†^, Mean ± SD, (n) | 0.33±0.82 (1) | 0.75±1.45 (6) | 0.705 |
| Dilation & aneurysm, n | 4 | 7 | 0.174 |
| Thickening, n | 1 | 17 | 0.078 |

Data shown as Mean ± SD if not informed. ESR, erythrocyte sedimentation rate; CRP, C-reactive protein; Indian Takayasu Clinical Activity Score. ^†^Number of involved arteries.
